# Supplementary material for: Brown bear communication hubs: patterns and correlates of tree rubbing and pedal marking at a long-term marking site
Source: PeerJ. 2021 Jan 29;9:e10447. doi: 10.7717/peerj.10447 (PMC7849508; doi:10.7717/peerj.10447)
Supplement: Table S1 — Camera days refer to the actual number of days the camera was working per month. [file peerj-09-10447-s002.docx]

**Table S1.** Complete monthly data set of bear visits to the marking site recorded by the camera trap. Camera days refer to the actual number of days the camera was working per month.

|  |  |  | Age/sex classes | | | | | **Total** |
| --- | --- | --- | --- | --- | --- | --- | --- | --- |
| **Year** | **Month** | **Camera days** | **Cubs** | **Females** | **Undetermined** | **Juveniles** | **Males** |  |
|  | April | 6 |  | 1 |  |  | 2 | 3 |
|  | May | 31 |  |  | 1 | 2 |  | 3 |
| 2012 | June | 30 |  | 1 |  |  | 2 | 3 |
|  | July | 4 |  |  |  |  |  |  |
|  | August | 0 |  |  |  |  |  |  |
|  | September | 3 |  |  |  |  | 1 | 1 |
|  | October | 31 | 3 | 7 | 1 | 2 | 8 | 21 |
|  | November | 30 | 6 | 5 |  | 1 | 5 | 17 |
|  | December | 16 | 6 | 2 |  | 1 |  | 9 |
|  | January | 0 |  |  |  |  |  |  |
|  | February | 0 |  |  |  |  |  |  |
|  | March | 11 |  |  |  |  | 3 | 3 |
|  | April | 30 |  | 1 |  |  | 10 | 11 |
|  | May | 31 |  | 6 | 1 |  | 13 | 20 |
| 2013 | June | 21 |  | 8 | 1 |  | 13 | 22 |
|  | July | 29 |  | 2 |  |  | 2 | 4 |
|  | August | 31 |  | 1 |  |  | 3 | 4 |
|  | September | 22 |  |  |  |  |  |  |
|  | October | 31 |  |  |  |  |  |  |
|  | November | 30 |  |  |  |  |  |  |
|  | December | 31 |  | 2 |  |  |  | 2 |
|  | January | 31 |  |  |  |  |  |  |
|  | February | 28 |  |  |  |  | 1 | 1 |
|  | March | 31 |  |  |  |  | 1 | 1 |
|  | April | 30 |  | 1 | 2 |  | 3 | 6 |
|  | May | 31 |  | 1 |  |  | 9 | 10 |
| 2014 | June | 30 |  |  | 2 |  | 3 | 5 |
|  | July | 31 |  | 1 | 1 |  | 4 | 6 |
|  | August | 31 | 5 | 1 |  |  | 4 | 10 |
|  | September | 30 |  | 3 |  |  | 4 | 7 |
|  | October | 31 | 16 | 5 | 4 |  | 3 | 28 |
|  | November | 30 | 8 | 2 | 7 |  |  | 17 |
|  | December | 31 |  |  |  |  |  |  |
|  | January | 31 |  |  |  |  |  |  |
|  | February | 28 |  |  |  |  |  |  |
|  | March | 31 |  |  |  |  | 1 | 1 |
|  | April | 30 |  | 1 | 1 |  | 13 | 15 |
|  | May | 31 |  | 3 | 1 | 6 | 10 | 20 |
| 2015 | June | 30 |  | 1 | 1 | 1 | 1 | 4 |
|  | July | 31 |  | 2 | 3 | 1 | 7 | 13 |
|  | August | 31 |  |  | 1 |  | 2 | 3 |
|  | September | 30 |  |  | 1 | 7 | 2 | 10 |
|  | October | 31 |  |  | 1 | 2 | 2 | 5 |
|  | November | 30 |  |  |  |  |  |  |
|  | December | 31 |  |  |  |  |  |  |
| Total |  | **1174** | **44** | **57** | **29** | **23** | **132** | **285** |
