# Supplementary material for: Brown bear communication hubs: patterns and correlates of tree rubbing and pedal marking at a long-term marking site
Source: PeerJ. 2021 Jan 29;9:e10447. doi: 10.7717/peerj.10447 (PMC7849508; doi:10.7717/peerj.10447)
Supplement: Table S2 [file peerj-09-10447-s003.docx]

**Table S2.** Number of visits of mammalian species attending the site or travelling through it between April 2013 and December 2015 as automatically recorded by the camera trap.

|  | Bear (*Ursus arctos*) | Wild boar (*Sus scrofa*) | Chamois (*Rupicapra pyrenaica*) | Roe Deer (*Capreolus capreolus*) | Fox (*Vulpes vulpes*) | Wildcat (*Felis silvestris*) | Genet (*Genetta genetta*) | Marten (*Martes martes*) | Squirrel (*Sciurus vulgaris*) | Wolf (*Canis lupus*) | Human (*Homo sapiens*) |
| --- | --- | --- | --- | --- | --- | --- | --- | --- | --- | --- | --- |
| ap-2013 | 10 | 1 | 3 | 1 |  | 1 |  |  |  |  |  |
| my-2013 | 15 | 2 | 1 | 2 |  | 1 |  |  |  |  |  |
| jn-2013 | 17 |  |  | 1 |  |  |  |  |  |  |  |
| jl-2013 | 4 |  |  |  |  | 1 |  |  |  |  |  |
| ag-2013 | 4 |  |  | 6 | 2 | 5 |  |  |  |  |  |
| sp-2013 |  | 2 |  | 2 |  | 1 |  | 1 |  |  |  |
| oc-2013 |  | 2 | 2 |  |  |  |  |  |  |  |  |
| nv-2013 |  |  | 3 | 3 |  | 1 |  |  |  |  | 1 |
| dc-2013 | 1 |  | 3 | 5 | 9 | 3 | 1 |  |  |  |  |
| ja-2014 | 1 | 1 | 6 | 1 | 2 |  |  |  |  |  |  |
| fb-2014 |  |  | 6 | 5 | 6 | 3 |  |  |  |  |  |
| mr-2014 | 1 | 3 | 1 | 4 |  | 1 |  | 1 |  |  |  |
| ap-2014 | 6 | 1 |  | 1 |  |  |  |  |  |  |  |
| my-2014 | 9 | 2 |  | 5 | 3 | 2 |  |  |  | 2 |  |
| jn-2014 | 5 |  |  |  |  |  |  |  |  |  |  |
| jl-2014 | 6 | 5 |  |  |  |  |  |  |  |  |  |
| ag-2014 | 6 |  | 2 |  |  |  | 3 |  |  |  |  |
| sp-2014 | 7 | 13 |  |  |  |  | 1 |  |  |  |  |
| oc-2014 | 15 | 6 |  | 1 | 2 |  | 1 |  |  |  |  |
| nv-2014 | 11 | 5 | 1 |  |  | 2 |  |  |  |  |  |
| dc-2014 |  | 7 | 2 |  | 2 |  |  |  |  |  |  |
| ja-2015 |  | 1 | 2 |  |  |  |  |  | 1 |  |  |
| fb-2015 |  | 1 | 19 |  |  |  |  |  |  |  |  |
| mr-2015 | 1 | 1 | 2 | 2 |  | 1 |  | 2 |  |  |  |
| ap-2015 | 16 |  |  | 1 | 1 |  | 1 |  |  |  |  |
| my-2015 | 13 |  |  | 2 | 1 |  |  | 1 | 1 |  |  |
| jn-2015 | 4 | 1 | 1 |  |  |  |  |  |  |  |  |
| jl-2015 | 11 | 3 | 1 |  |  | 1 |  |  |  |  |  |
| ag-2015 | 3 |  |  |  |  | 1 |  |  |  |  |  |
| sp-2015 | 7 | 8 | 1 | 1 | 1 |  |  |  |  |  |  |
| oc-2015 | 5 | 5 | 1 | 1 |  |  |  |  |  |  |  |
| nv-2015 |  | 1 | 1 |  | 1 | 1 |  |  |  |  |  |
| dc-2015 |  |  | 3 |  | 3 |  |  |  |  |  |  |
|  | 178 | 71 | 61 | 44 | 33 | 25 | 7 | 5 | 2 | 2 | 1 |
