# Supplementary material for: Brown bear communication hubs: patterns and correlates of tree rubbing and pedal marking at a long-term marking site
Source: PeerJ. 2021 Jan 29;9:e10447. doi: 10.7717/peerj.10447 (PMC7849508; doi:10.7717/peerj.10447)
Supplement: Table S3 [file peerj-09-10447-s004.docx]

**Table S3.** Results of the models within ΔAIC < 2 and the null and full models for each set of analyses.

| **response: bear visit in a given day (0,1)** | | | |  |  |  |  |  |  |  |  |
| --- | --- | --- | --- | --- | --- | --- | --- | --- | --- | --- | --- |
| model | df | Intercept | days_male | season |  |  |  |  | AICc | delta | W-AICc |
| Full | 4 | 1.139 | -1.823 | -0.379 |  |  |  |  | 930.3 | 0.00 | 1.00 |
| Null | 2 |  |  |  |  |  |  |  | 1093.6 |  |  |
| **response: sniff pedal marks in a given visit (0,1)** | | | |  |  |  |  |  |  |  |  |
| models | df | Intercept | Prec_pedal | Temp_pedal | season | days_pedal | days | age_sex | AICc | delta | W-AICc |
| 1 | 6 | -2.069 | -0.013 | -0.011 | 2.046 | -0.725 |  |  | 195.1 | 0.00 | 0.33 |
| 2 | 6 | -1.881 | -0.014 | -0.012 | 1.830 |  | -2.667 |  | 195.9 | 0.85 | 0.21 |
| 3 | 5 | -2.091 | -0.013 | -0.010 | 1.617 |  |  |  | 196.5 | 1.41 | 0.16 |
| 4 | 7 | -1.961 | -0.013 | -0.012 | 2.069 | -0.544 | -0.424 |  | 196.6 | 1.50 | 0.15 |
| 5 | 9 | -2.667 | -0.014 | -0.012 | 2.195 | -0.923 |  | + | 196.7 | 1.65 | 0.14 |
| Full | 10 |  |  |  |  |  |  |  | 198.8 |  |  |
| Null | 2 |  |  |  |  |  |  |  | 204.7 |  |  |
| **response: pedal marking in a given visit (0,1)** | | | |  |  |  |  |  |  |  |  |
| model | df | Intercept | tree_rubbing | days_pedal | sniff_pedal | season |  |  | AICc | delta | W-AICc |
| 1 | 4 | 1.946 | 1.315 | -1.255 |  |  |  |  | 117.1 | 0.00 | 0.50 |
| 2 | 5 | 1.802 | 1.362 | -1.235 | 0.566 |  |  |  | 118.5 | 1.34 | 0.26 |
| 3 | 5 | 1.416 | 1.407 | -1.423 |  | 0.445 |  |  | 118.6 | 1.48 | 0.24 |
| Full | 7 |  |  |  |  |  |  |  | 120.2 |  |  |
| Null | 2 |  |  |  |  |  |  |  | 126.3 |  |  |
| **response: sniff tree in a given visit (0,1)** | | | |  |  |  |  |  |  |  |  |
| models | df | Intercept | days | Prec_tree | age_sex | season | Temp_tree |  | AICc | delta | W-AICc |
| 1 | 4 | -0.090 | 0.885 | -0.011 |  |  |  |  | 249.3 | 0.00 | 0.31 |
| 2 | 7 | -0.105 | 0.976 | -0.010 | + |  |  |  | 249.6 | 0.26 | 0.27 |
| 3 | 5 | 0.288 | 0.912 | -0.013 |  |  | -0.027 |  | 250.8 | 1.48 | 0.15 |
| 4 | 6 | -0.325 | 0.809 |  | + |  |  |  | 251.0 | 1.65 | 0.14 |
| 5 | 5 | 0.145 | 0.922 | -0.011 |  | -0.184 |  |  | 251.1 | 1.78 | 0.13 |
| Full | 13 |  |  |  |  |  |  |  | 258.3 |  |  |
| Null | 2 |  |  |  |  |  |  |  | 253.4 |  |  |
| **response: tree rubbing in a given visit (0,1)** | | | |  |  |  |  |  |  |  |  |
| models | df | Intercept | pedal marking | sniff_tree | days_tree | age_sex |  |  | AICc | delta | W-AICc |
| 1 | 8 | -3.610 | 1.293 | 1.412 | 0.857 | + |  |  | 230.3 | 0.00 | 0.48 |
| 2 | 5 | -2.959 | 1.679 | 1.408 | 0.940 |  |  |  | 231.1 | 0.80 | 0.32 |
| 3 | 7 | -3.099 | 1.247 | 1.504 |  | + |  |  | 232.0 | 1.73 | 0.20 |
| Full | 10 |  |  |  |  |  |  |  | 232.4 |  |  |
| Null | 2 |  |  |  |  |  |  |  | 278.6 |  |  |
