# Supplementary material for: Brown bear communication hubs: patterns and correlates of tree rubbing and pedal marking at a long-term marking site
Source: PeerJ. 2021 Jan 29;9:e10447. doi: 10.7717/peerj.10447 (PMC7849508; doi:10.7717/peerj.10447)
Supplement: Table S4 [file peerj-09-10447-s005.docx]

**Table S4.** Number of behaviors displayed by bears per month as recorded by the camera trap at the marking site.

|  | Pedal marking | Tree rubbing | Sniffing pedal mark | Sniffing tree | Other | Total |
| --- | --- | --- | --- | --- | --- | --- |
| January |  |  |  |  |  |  |
| February |  |  |  |  | 1 | 1 |
| March | 5 | 1 | 1 | 4 |  | 11 |
| April | 23 | 21 | 8 | 28 | 2 | 82 |
| May | 29 | 17 | 5 | 23 | 11 | 85 |
| June | 17 | 11 | 7 | 14 | 9 | 58 |
| July | 11 | 8 | 3 | 12 | 4 | 38 |
| August | 7 | 6 | 2 | 12 | 3 | 30 |
| September | 3 | 6 | 6 | 11 | 4 | 30 |
| October | 13 | 10 | 18 | 31 | 11 | 83 |
| November | 5 | 12 | 7 | 13 | 13 | 50 |
| December |  | 4 | 1 | 5 | 4 | 14 |
| Total | 113 | 96 | 58 | 153 | 62 | 482 |
