# Supplementary material for: Brown bear communication hubs: patterns and correlates of tree rubbing and pedal marking at a long-term marking site
Source: PeerJ. 2021 Jan 29;9:e10447. doi: 10.7717/peerj.10447 (PMC7849508; doi:10.7717/peerj.10447)
Supplement: Table S5 [file peerj-09-10447-s006.docx]

**Table S5.** Number of behaviors displayed by bears per age-sex class as recorded by the camera trap at the marking site.

|  | Pedal marking | Tree rubbing | Sniffing pedal mark | Sniffing tree | Other | Total |
| --- | --- | --- | --- | --- | --- | --- |
| Females | 0 | 5 | 12 | 29 | 20 | 66 |
| Males | 107 | 63 | 30 | 78 | 5 | 283 |
| Juveniles | 4 | 9 | 9 | 11 | 7 | 40 |
| Cubs | 0 | 15 | 3 | 27 | 12 | 57 |
| Undetermined | 2 | 4 | 4 | 8 | 18 | 36 |
| Total | 113 | 96 | 58 | 153 | 62 |  |
