# Supplementary material for: Brown bear communication hubs: patterns and correlates of tree rubbing and pedal marking at a long-term marking site
Source: PeerJ. 2021 Jan 29;9:e10447. doi: 10.7717/peerj.10447 (PMC7849508; doi:10.7717/peerj.10447)
Supplement: Table S6 [file peerj-09-10447-s007.docx]

**Table S6.** Visits of individually recognized adult male bears recorded at the marking site by the camera.

|  | | M1 (Bertino) | M2 (Cornualles) | M3 (Tifus) | M4 (Xanuco) |
| --- | --- | --- | --- | --- | --- |
| 2012 | Apr | 1 | 1 |  |  |
|  | May |  |  |  |  |
|  | Jun |  |  |  |  |
|  | Sep |  | 1 |  |  |
|  | Oct |  | 1 | 6 |  |
|  | Nov | 1 |  | 4 |  |
|  | Dec |  |  |  |  |
|  | total | 2 | 3 | 10 |  |
| 2013 | Mar |  |  | 3 |  |
|  | Apr | 2 | 1 | 3 |  |
|  | May | 2 | 2 | 2 |  |
|  | Jun |  | 7 | 2 |  |
|  | Jul |  |  | 1 |  |
|  | Aug |  | 2 | 1 |  |
|  | Dec |  |  |  |  |
|  | total | 4 | 12 | 12 |  |
| 2014 | Feb |  |  |  |  |
|  | Mar |  | 1 |  |  |
|  | Apr | 1 | 1 |  |  |
|  | May | 2 |  | 4 |  |
|  | Jun |  |  | 1 |  |
|  | Jul |  | 1 | 2 |  |
|  | Aug | 1 | 1 |  |  |
|  | Sep |  | 1 |  |  |
|  | Oct | 1 |  |  |  |
|  | Dec |  |  |  |  |
|  | total | 5 | 5 | 7 |  |
| 2015 | Mar | 1 |  |  |  |
|  | Apr |  | 6 |  | 5 |
|  | May | 1 |  | 1 | 2 |
|  | Jun |  | 1 |  |  |
|  | Jul |  | 5 |  |  |
|  | Aug |  | 1 |  | 1 |
|  | Sep |  |  |  | 2 |
|  | Oct |  | 2 |  |  |
|  | total | 2 | 15 | 1 | 10 |
| TOTAL | Feb |  |  |  |  |
|  | Mar | 1 | 1 | 3 |  |
|  | Apr | 4 | 9 | 3 | 5 |
|  | May | 5 | 2 | 7 | 2 |
|  | Jun |  | 8 | 3 |  |
|  | Jul |  | 6 | 3 |  |
|  | Aug | 1 | 4 | 1 | 1 |
|  | Sep |  | 2 |  | 2 |
|  | Oct | 1 | 3 | 6 |  |
|  | Nov | 1 |  | 4 |  |
|  | Dec |  |  |  |  |
|  | TOTAL | **13** | **35** | **30** | **10** |
