# Supplementary material for: Brown bear communication hubs: patterns and correlates of tree rubbing and pedal marking at a long-term marking site
Source: PeerJ. 2021 Jan 29;9:e10447. doi: 10.7717/peerj.10447 (PMC7849508; doi:10.7717/peerj.10447)
Supplement: Table S7 [file peerj-09-10447-s008.docx]

**Table S7.** Number of different behaviors displayed by individually recognized adult male bears recorded at the marking site by the camera trap; same codes as Table S6.

| Individual | Pedal marking | Sniff pedal | Tree rubbing | Sniff tree | Total |
| --- | --- | --- | --- | --- | --- |
| M1 (Bertino) | 1 | 1 | 5 | 13 | 13 |
| M2 (Cornualles) | 34 | 8 | 27 | 18 | 35 |
| M3 (Tifus) | 29 | 9 | 6 | 14 | 30 |
| M4 (Xanuco) | 7 | 1 | 6 | 9 | 10 |
| Total | 71 | 19 | 44 | 54 | 88 |
