# Supplementary material for: Brown bear communication hubs: patterns and correlates of tree rubbing and pedal marking at a long-term marking site
Source: PeerJ. 2021 Jan 29;9:e10447. doi: 10.7717/peerj.10447 (PMC7849508; doi:10.7717/peerj.10447)
Supplement: Table S8 — Models were GLMMs with binomial distribution and year and id as random factors (all unidentified individuals were pooled into a single id level and considered as the same individual). The models on pedal marking were run only on males and the rest with all types of individuals except for cubs. See Table 1 for a description of the variables. [file peerj-09-10447-s009.docx]

**Table S8. Estimates of the effect of the factors included in the best models from Table 3 but including individual ID as an additional random factor.** Models were GLMMs with binomial distribution and year and id as random factors (all unidentified individuals were pooled into a single id level and considered as the same individual). The models on pedal marking were run only on males and the rest with all types of individuals except for cubs. See Table 1 for a description of the variables.

| **Model** | **Estimate** | **SE** | ***p*** |
| --- | --- | --- | --- |
| ***sniff pedal marks*** (all classes of individuals except cubs) | | | |
| (intercept) | -2.069 | 0.797 | 0.009 |
| days_pedal | -0.725 | 0.389 | 0.062 |
| Prec_pedal | -0.013 | 0.006 | 0.036 |
| Temp_pedal | -0.011 | 0.005 | 0.013 |
| season | 2.046 | 0.546 | <0.001 |
| R^2^ (marg) = 0.21 |  |  |  |
| R^2^ (cond) = 0.26 |  |  |  |
| ***pedal marking*** (males) | | | |
| (intercept) | 2.394 | 0.910 | 0.009 |
| days_pedal | -1.008 | 0.577 | 0.081 |
| tree_rubbing | 1.527 | 0.618 | 0.013 |
| R^2^ (marg) = 0.13 |  |  |  |
| R^2^ (cond) = 0.49 |  |  |  |
| ***sniff tree*** (all classes of individuals except cubs) | | | |
| (intercept) | 0.585 | 0538 | 0.277 |
| days | 0.891 | 0.404 | 0.028 |
| Prec_tree | -0.015 | 0.006 | 0.015 |
| R^2^ (marg) = 0.06 |  |  |  |
| R^2^ (cond) = 0.32 |  |  |  |
| ***tree rubbing*** (all classes of individuals except cubs) | | | |
| (intercept) | -5.198 | 1.127 | <0.0001 |
| days_tree | 0.909 | 0.452 | 0.044 |
| sniff_tree | 1.985 | 0.449 | <0.0001 |
| pedal_marking | 1.511 | 0.597 | 0.011 |
| age_sex |  |  |  |
| Undetermined | 0.069 | 0.911 | 0.446 |
| Juvenile | 2.297 | 0.867 | 0.008 |
| Male | 1.661 | 0.841 | 0.048 |
| R^2^ (marg) = 0.33 |  |  |  |
| R^2^ (cond) = 0.66 |  |  |  |
