# Supplementary material for: Brown bear communication hubs: patterns and correlates of tree rubbing and pedal marking at a long-term marking site
Source: PeerJ. 2021 Jan 29;9:e10447. doi: 10.7717/peerj.10447 (PMC7849508; doi:10.7717/peerj.10447)
Supplement: Table S9 — Approximation of the minimum number of individuals visiting the site per year. Four of the males (Cornualles; Tifus; Bertino and Xanuco) visited the site on multiple occasions (see Tables S6 and S7 and section Identification of individualized bears in this Supplementary Material). [file peerj-09-10447-s010.docx]

**Table S9.** Minimum number of individuals visiting the site per year. Approximation to the estimate of the minimum number of individuals visiting the site per year. Four of the males (*Cornualles*; *Tifus*; *Bertino and Xanuco*) visited the site on multiple occasions (see Tables S6 and S7 and section Identification of individualized bears in this Supplementary Material).

| Sex-age class |  |  | Minimum number within class |
| --- | --- | --- | --- |
| Males | 2012 | *Cornualles*; *Tifus*; *Bertino*; plus 1 more visit of an unknown male | 4 |
|  | 2013 | *Cornualles*; *Tifus*; *Bertino*; *Negrito* (2 visits); *Hoyuelo* (3 visits); *Lunares* (1 visit); plus 10 more visits of unknown males | 7 |
|  | 2014 | *Cornualles*; *Tifus*; *Bertino*; *Rubio* (2 visits); *Lunares* (2 visits); *Culo* *seco* (1 visit); plus 9 more visits of unknown males | 6 |
|  | 2015 | *Cornualles*; *Tifus*; *Bertino*; *Xanuco*; *Xiron* (1 visit); plus 9 more visits of unknown males | 6 |
| Females | 2012 | *Güesi*; one female with 3 cubs of the year; 2 visits of unknown females | 3 |
|  | 2013 | *Güesi*; plus 19 more visits of unknown females | 2 |
|  | 2014 | *Güesi*; at least one female with 3 cubs of the year; plus 4 more visits of unknown females | 3 |
|  | 2015 | *Güesi*; one female with 2 cubs of second year; plus 4 more visits of unknown females | 3 |
| Cubs (first year) and second year with mother | 2012 | 3 cubs of the year | 3 |
|  | 2013 |  |  |
|  | 2014 | 3 cubs of the year | 3 |
|  | 2015 | 2 of second year | 2 |
| Independent young (second and third year) | 2012 | 3 independent siblings of third year together; 1 third year solitary (multiple visits)* | 3 |
|  | 2013 |  |  |
|  | 2014 |  |  |
|  | 2015 | 2 independent siblings of second year together (multiple visits); 3 independent siblings of third year together (multiple visits); 1 third year solitary (multiple visits) | 6 |
| Undetermined | 2012 | 1 characteristic individual of unknown sex; plus 2 more visits | 2 |
|  | 2013 | 1 characteristic individual of unknown sex; plus 2 more visits | 2 |
|  | 2014 | 4 visits of a bicolor individual of unknown sex; 1 characteristic individual of unknown sex; plus 12 more visits | 3 |
|  | 2015 | 9 visits | 1 |

*as the observations of lone individuals occurred after the observations of the group, we cannot rule out they were not the same
