# Supplementary figures and images for: Brown bear communication hubs: patterns and correlates of tree rubbing and pedal marking at a long-term marking site
Source: PeerJ. 2021 Jan 29;9:e10447. doi: 10.7717/peerj.10447 (PMC7849508; doi:10.7717/peerj.10447)

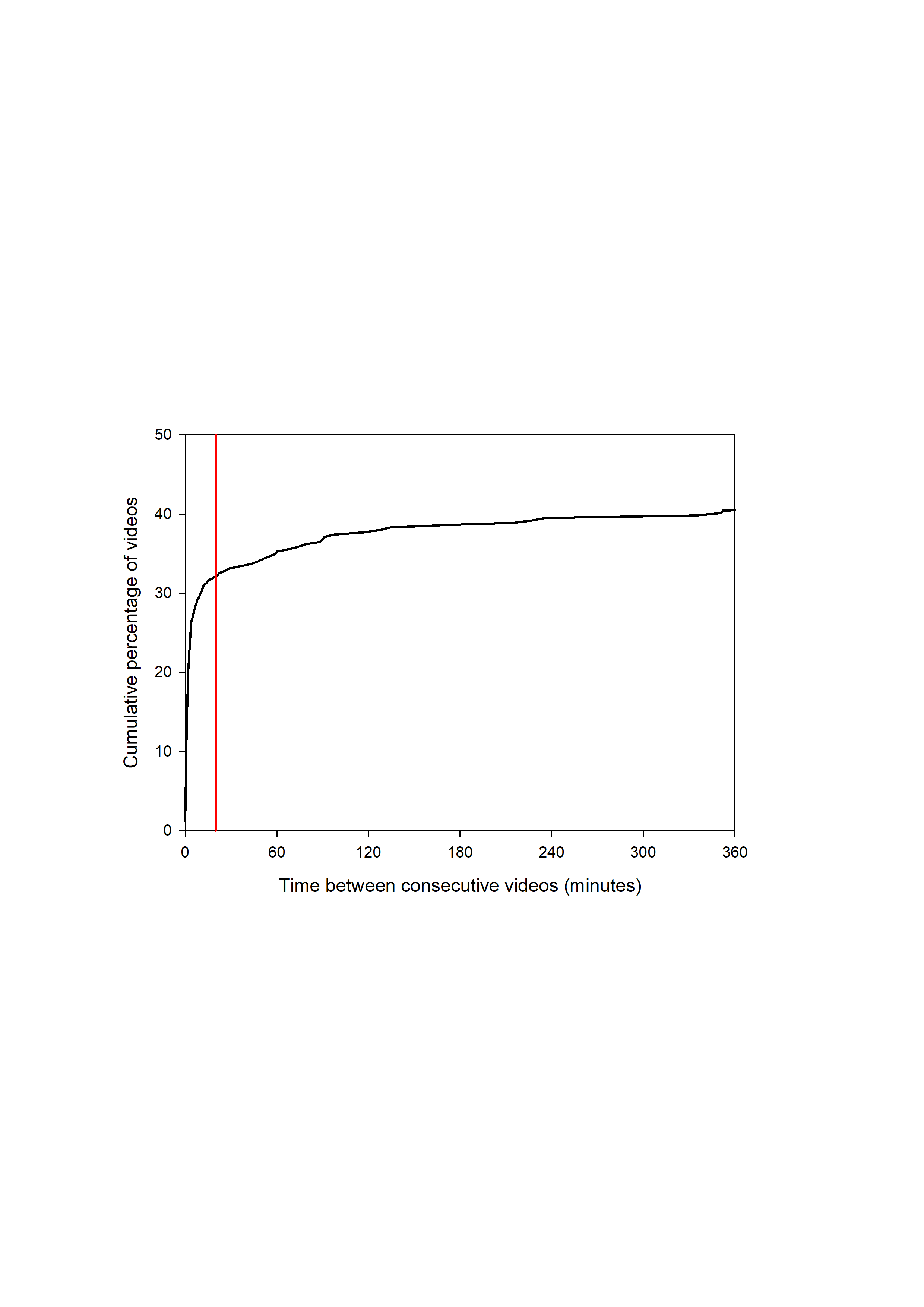

Supplement: Figure S1 — All videos separated by 20 minutes (red line threshold) or less were considered as part of the same visit event. [file peerj-09-10447-s011.png]

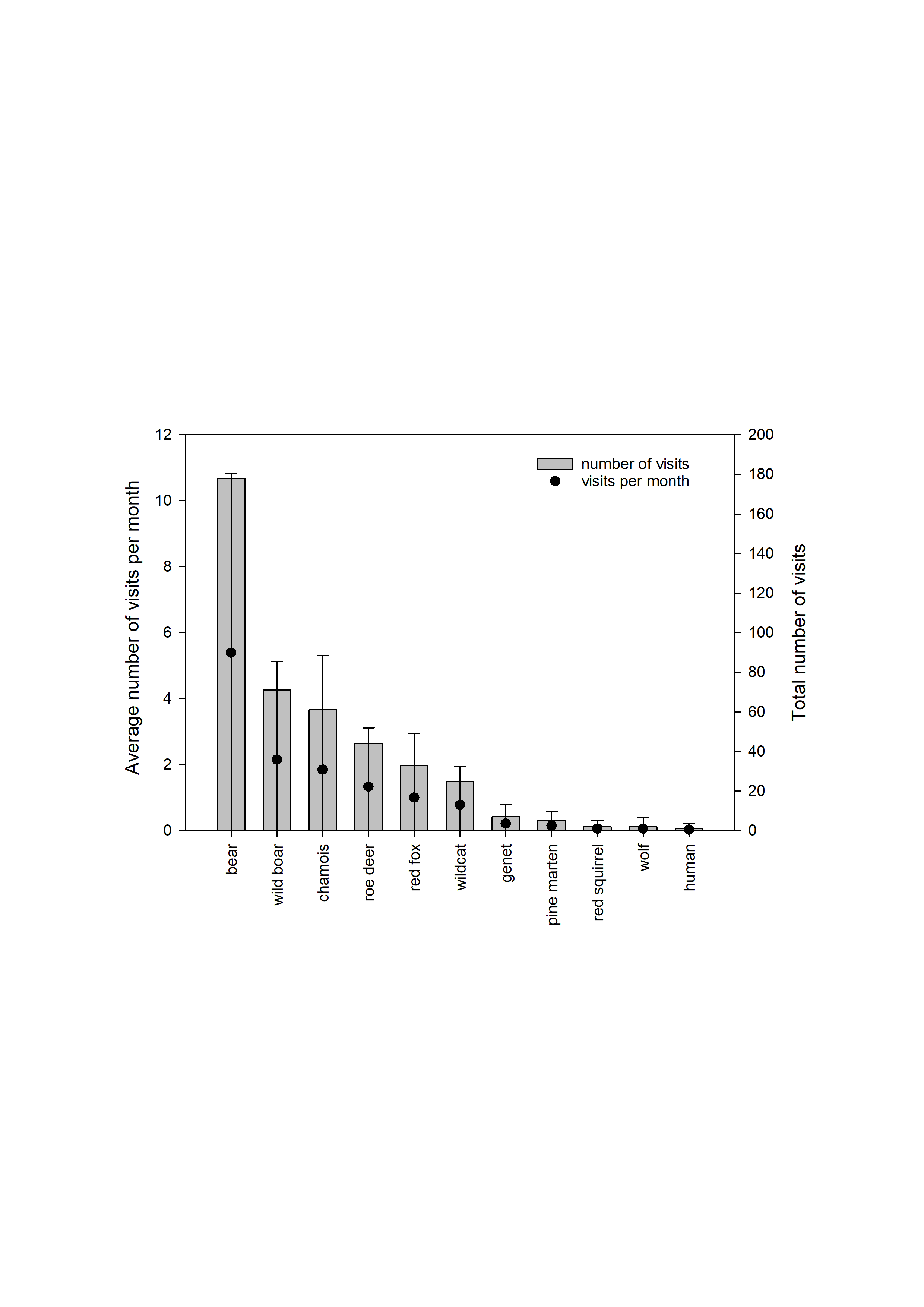

Supplement: Figure S2 — Each visit defined by the group of videos of a species that was separated by more than 20 minutes to the nearest video (grey bars, right axis) and average number of visits per month (± standard deviation; black dots, left axis). Plotted data refer to the subperiod between April 2013 and December 2015 (Table S1). Wild boar Sus scrofa, chamois Rupicapra pyrenaica, roe deer Capreolus capreolus, red fox Vulpes vulpes, wildcat Felis sylvestris, common genet Genetta genetta, pine marten Martes martes, red squirrel Sciurus vulgaris, wolf Canis lupus and human Homo sapiens. [file peerj-09-10447-s012.png]

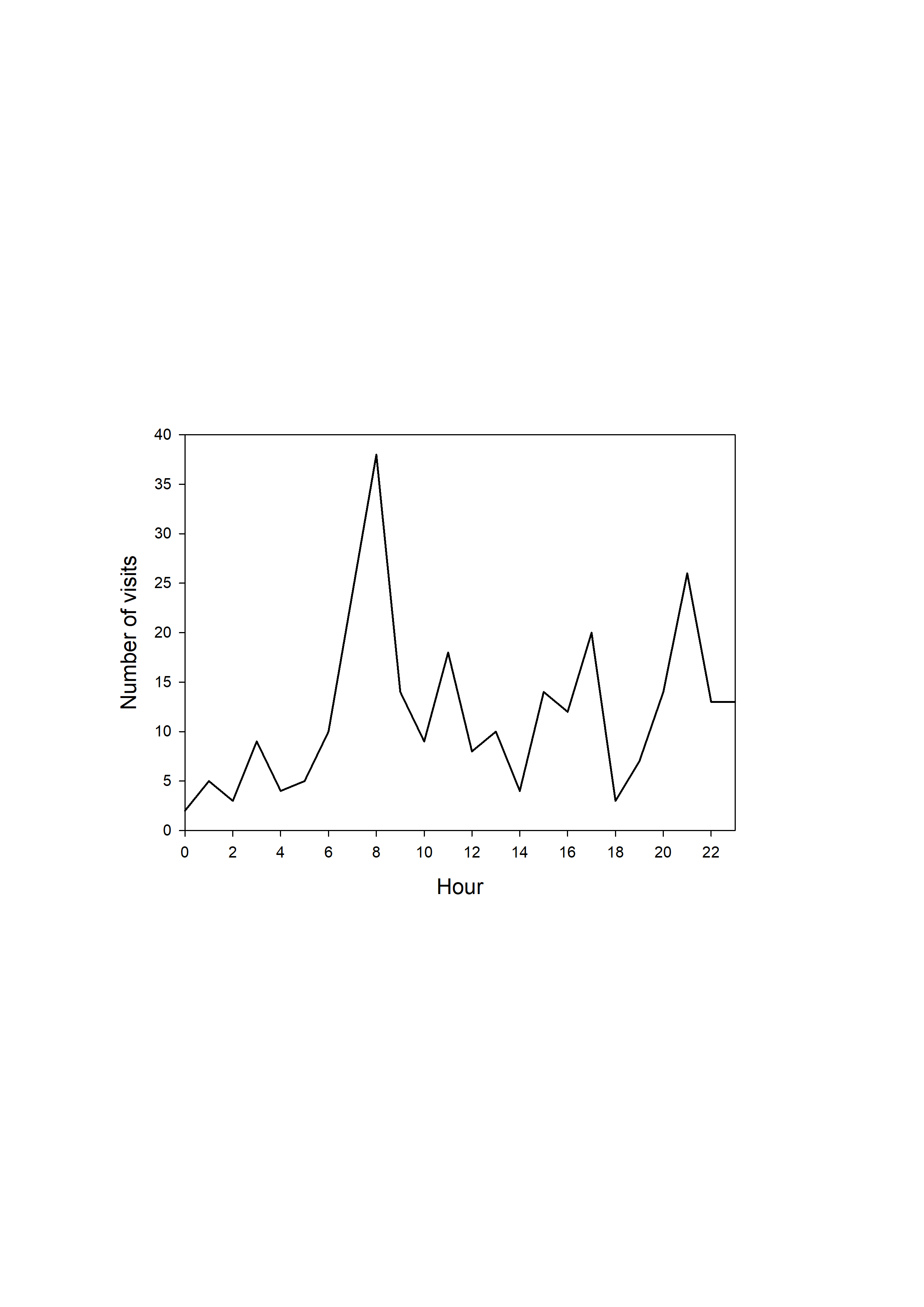

Supplement: Figure S3 [file peerj-09-10447-s013.png]
