# Supplementary material for: Brown bear communication hubs: patterns and correlates of tree rubbing and pedal marking at a long-term marking site
Source: PeerJ. 2021 Jan 29;9:e10447. doi: 10.7717/peerj.10447 (PMC7849508; doi:10.7717/peerj.10447)

**Identification of individualized bears**

The brown bear population in the Cantabrian Mountains is special since individuals are very easy to observe. In this mountain area, the steep slopes and the low forest cover make it easy to see bears or even count the number of animals in a valley by scanning the area with spotting scopes from vantage points. The annual count of the number of females with cubs of the year is the long-term method used to census this population. The professional technicians doing those censuses are experts in recognizing the sex and age of individuals; they do it every day as a job. Our co-authors who did the extraction of data from the videos (DR and AFG, and JN to a lesser extent) belong to this group of experts. The data obtained for the censuses is unbiased when compared with estimates from genetic capture-recapture studies (eg. Pérez et al 2014). Many of the individuals living in the subpopulation where the sampling site is located are known due to the intense observation-based monitoring. As a consequence, when animals appear in the videos, in many occasions, it is possible to identify the individuals, especially in the case of characteristic adult males. When we extracted the data on sex and age in the case of the few doubtful cases we crosschecked the assignation between authors. In case of disagreement or doubt the animals were assigned to the class “undertermined” (most of which would be animals in their third year of life and of unknown sex).

Bears in the Cantabrian Mountains have another special characteristic: the number of individuals carrying unique marks in their coat is large. Apart from the typical scars that adult males have, many animals have a whitish collar around their neck with variable shape (resembling the one that yearling bears typically have). This allows the identification of these individuals. We have been very careful and only assigned individual id to unmistakable animals. We were able to identify several other individuals but with more uncertainty and therefore we did not consider those in the description. Note that individual bears are known due to their repeated observations in the field and not only from appearing in the camera recordings. We took a conservative approach and only describe the use and behaviors of the four most commonly observed males, pooling all the others in their sex age classes (which include a group for undetermined individuals). Here we provide a description and example images (extracted from videos or obtained by diggiscoping in other sites of the area) of these four males (M1 to M4) and a characteristic female (F1) that appeared repeatedly.

*M1 (Bertino)*

A mid-sized adult male observed in 2011 for the first time as a young adult. Body color is light chocolate brown, with a golden face and very conspicuous marks on both sides of the neck that are connected on the nape by a wide band forming a kind of semi-collar. Right and left sides of the collar are quite different in size and shape, the left one being slightly bigger and more corrugated.


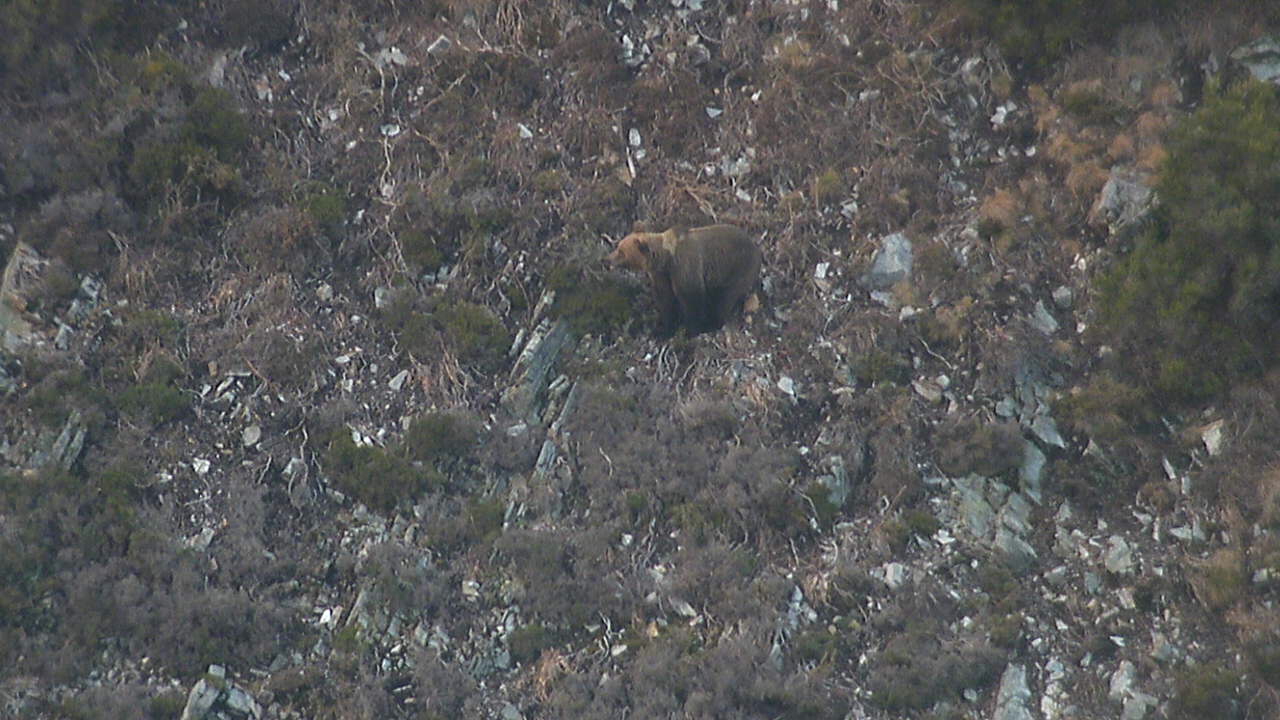

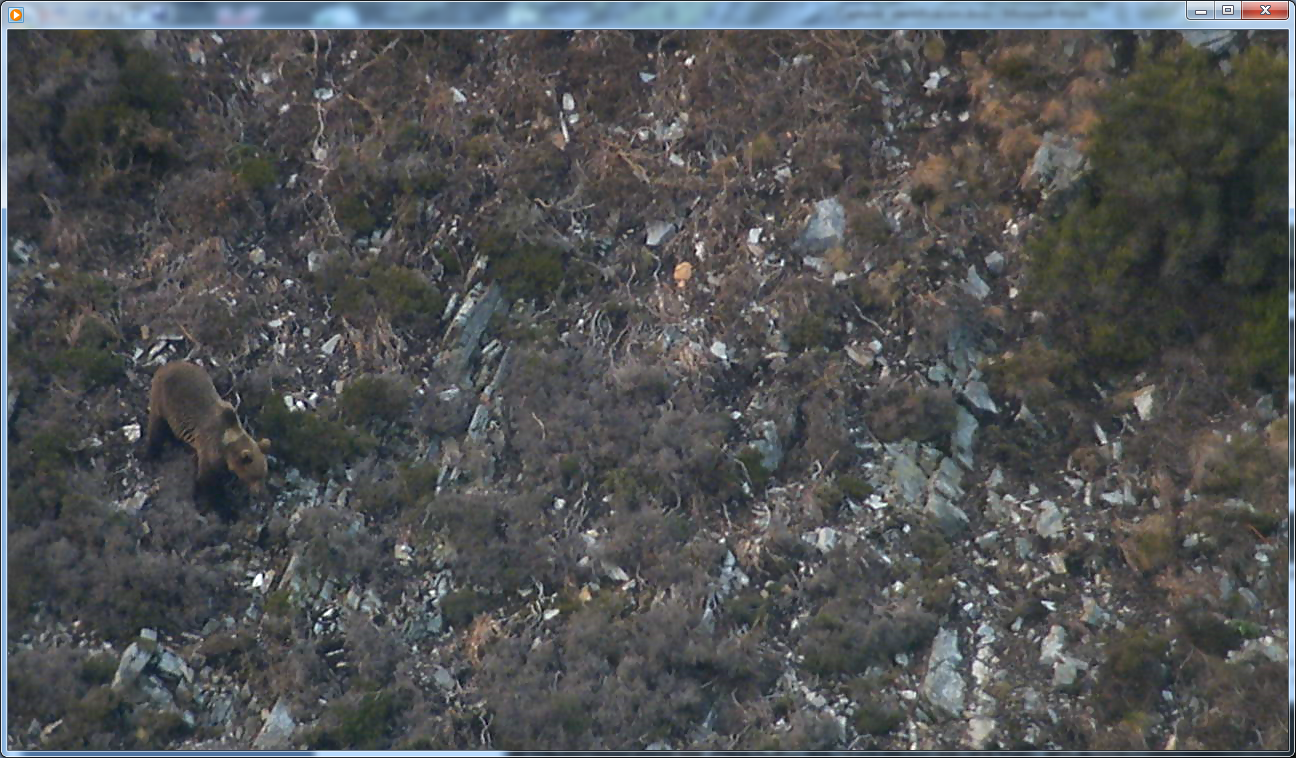

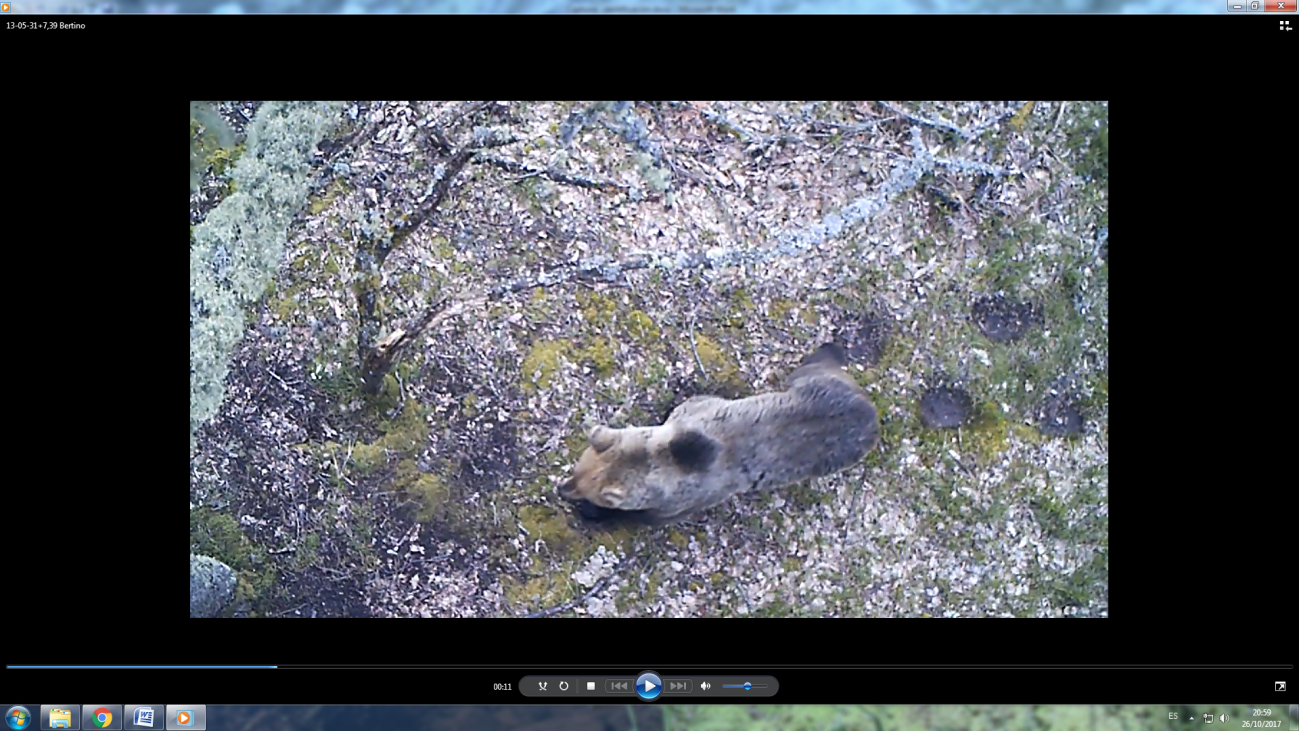


*M2 (Cornualles)*

A big adult male observed in the area in 2007 for the first time as an inmature bear, seemingly in their third or fourth calendar year. Body color is dark brown, and wears highly conspicuous markings on both sides of the neck that are connected in the nape by a narrow band, forming a semi-collar. The right side of the neck markings is larger than the left one, being this one more complex in shape. The connection of the markings in the neck wears a dark spot that make this bear unmistakable.


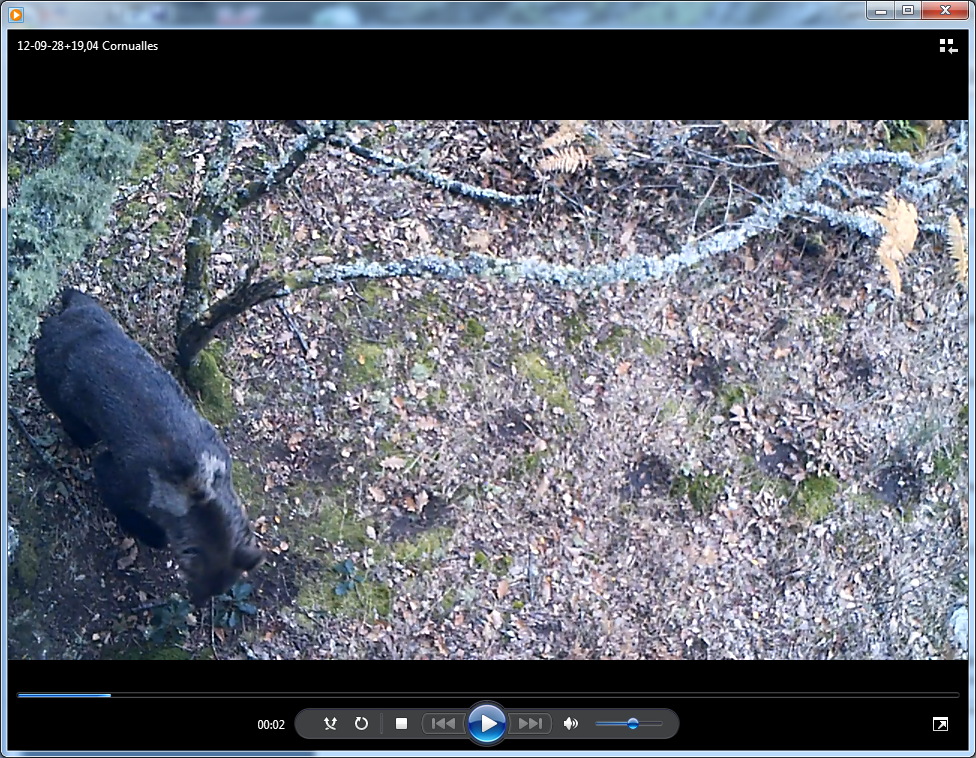

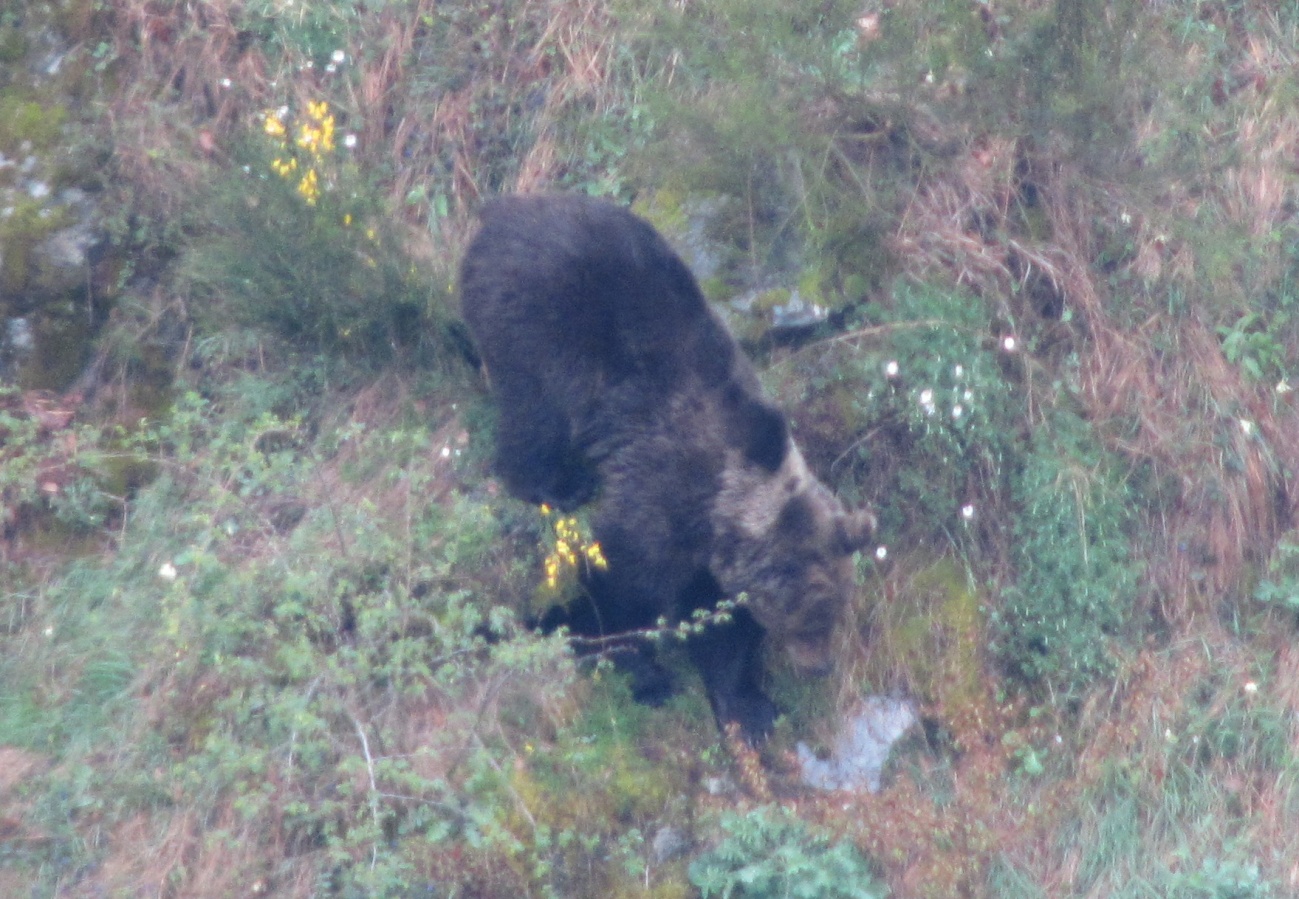

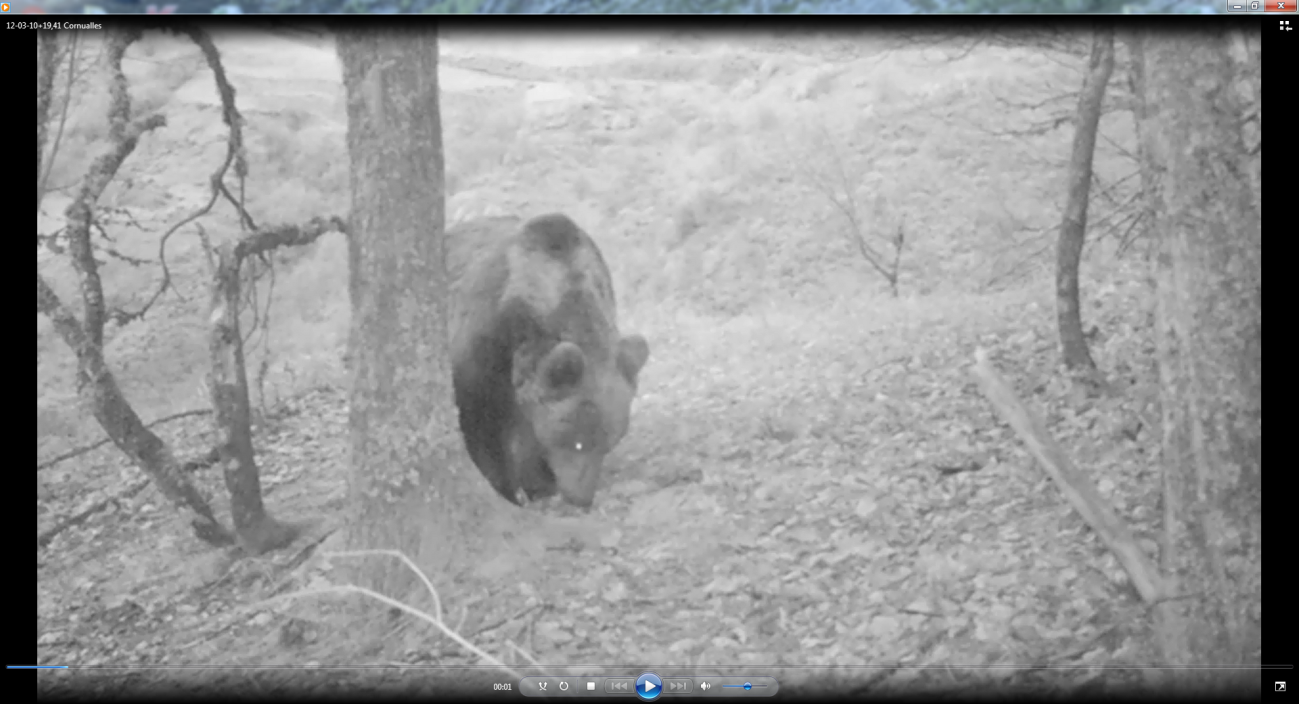


*M3 (Tifus)*

A quite big, powerful and impressing adult male. Body color is light grizzled brown. It has very conspicuous neck markings unconnected through the nape or throat. Neck marks are bright golden-yellow, the one on the right with a triangular shape, and the left one with a rhomboidal shape. M3 was well known in the area and performed many of the mating events witnessed along the study period in the surroundings (several sq. km) of the study area.


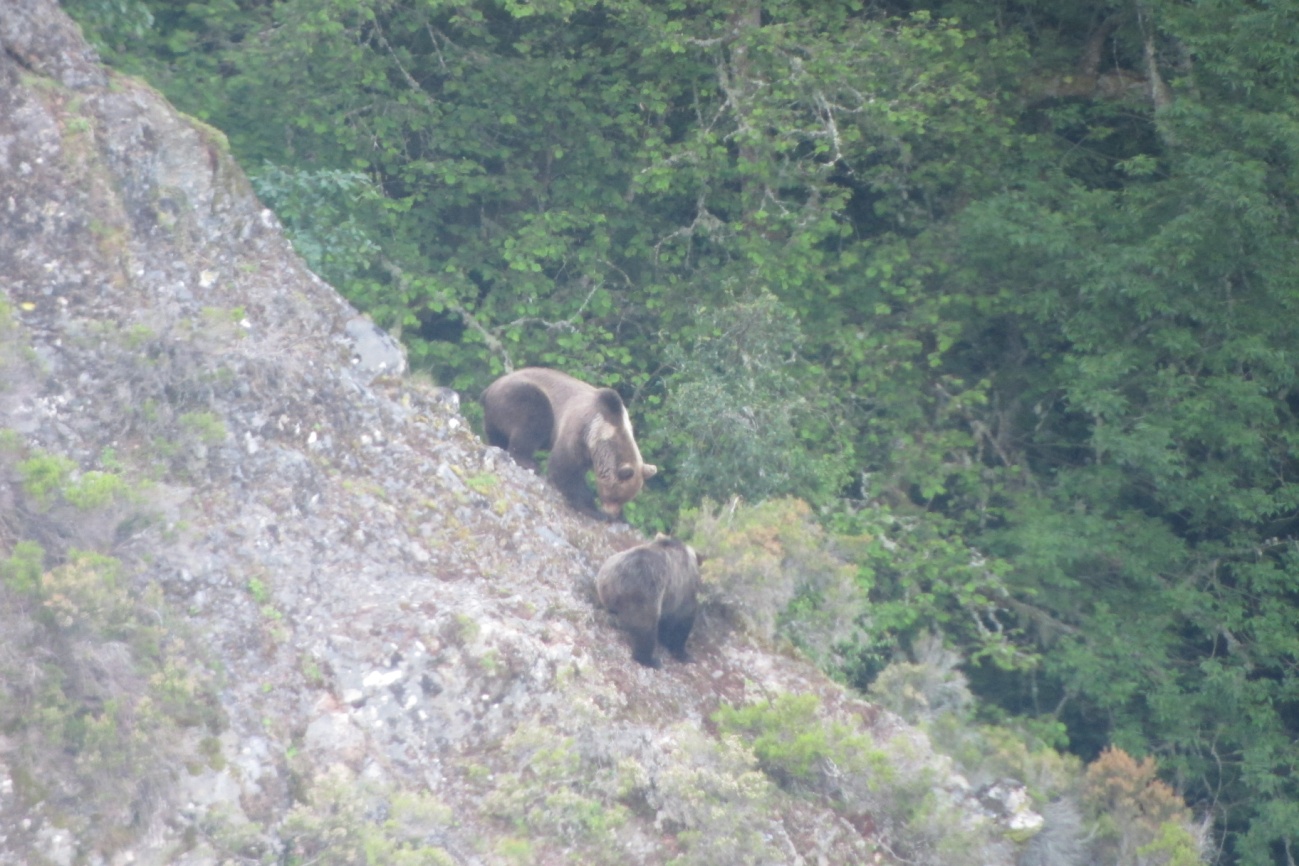

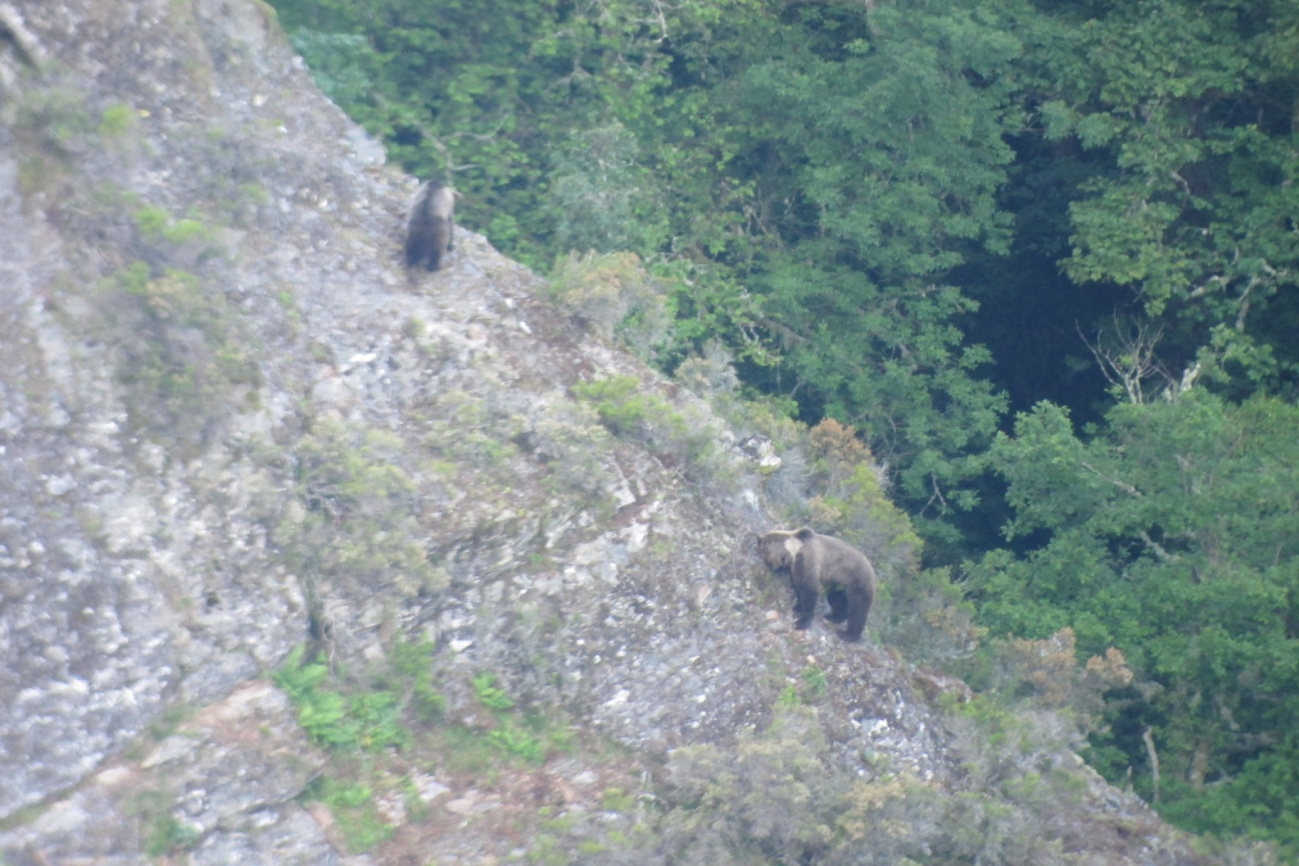

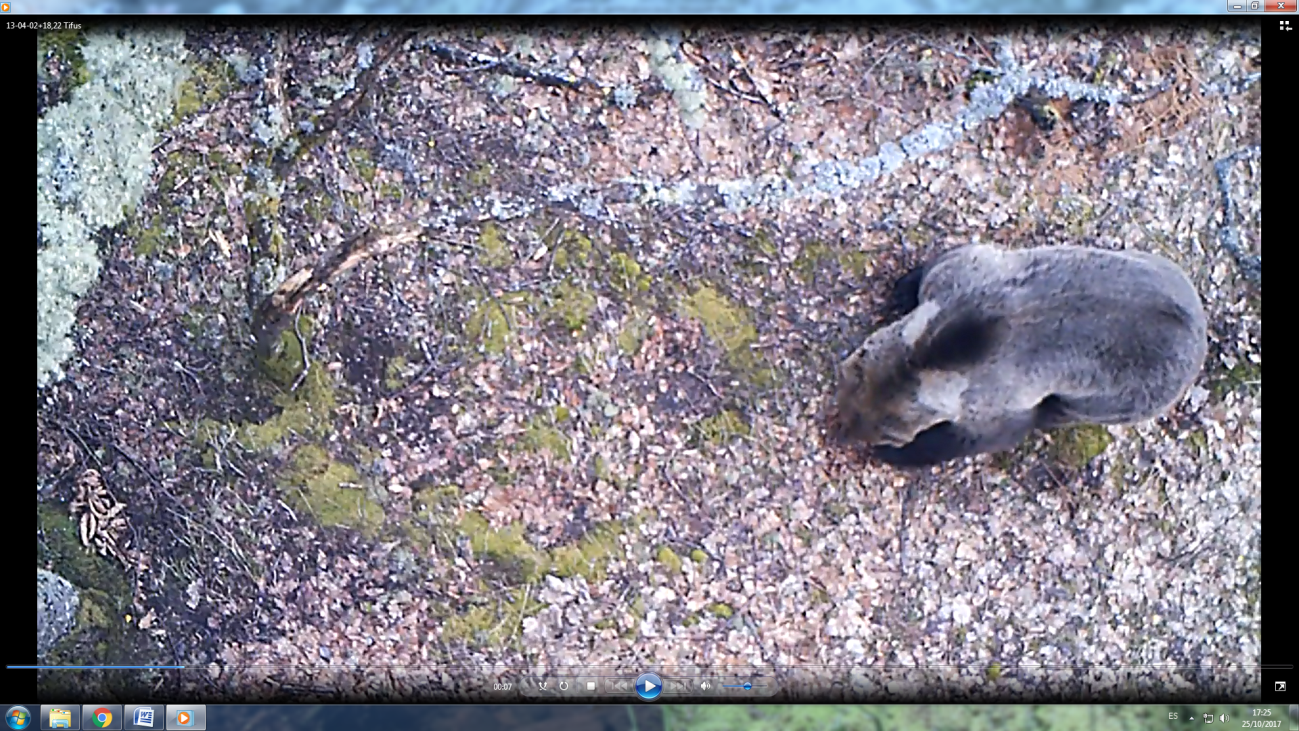

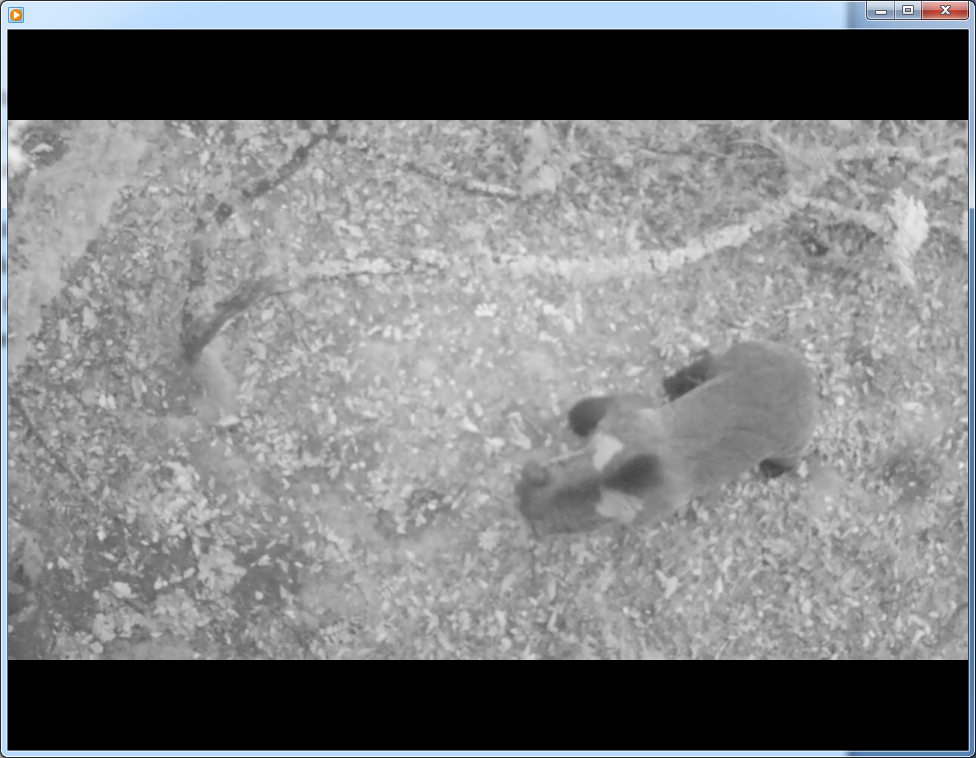


*M4 (Xanuco)*

A mid-sized male bear, first recorded at the end of the study period, during 2015, seemingly as a young adult. Body color is dark brown but with grizzled shadings along the shoulders. He wears small but conspicuous (at least at the camera range) light neck markings on both sides, almost symmetrical in position and shape; the one of the right side is an almost perfect vertical rectangle that allowed identification on most recordings at the camera.


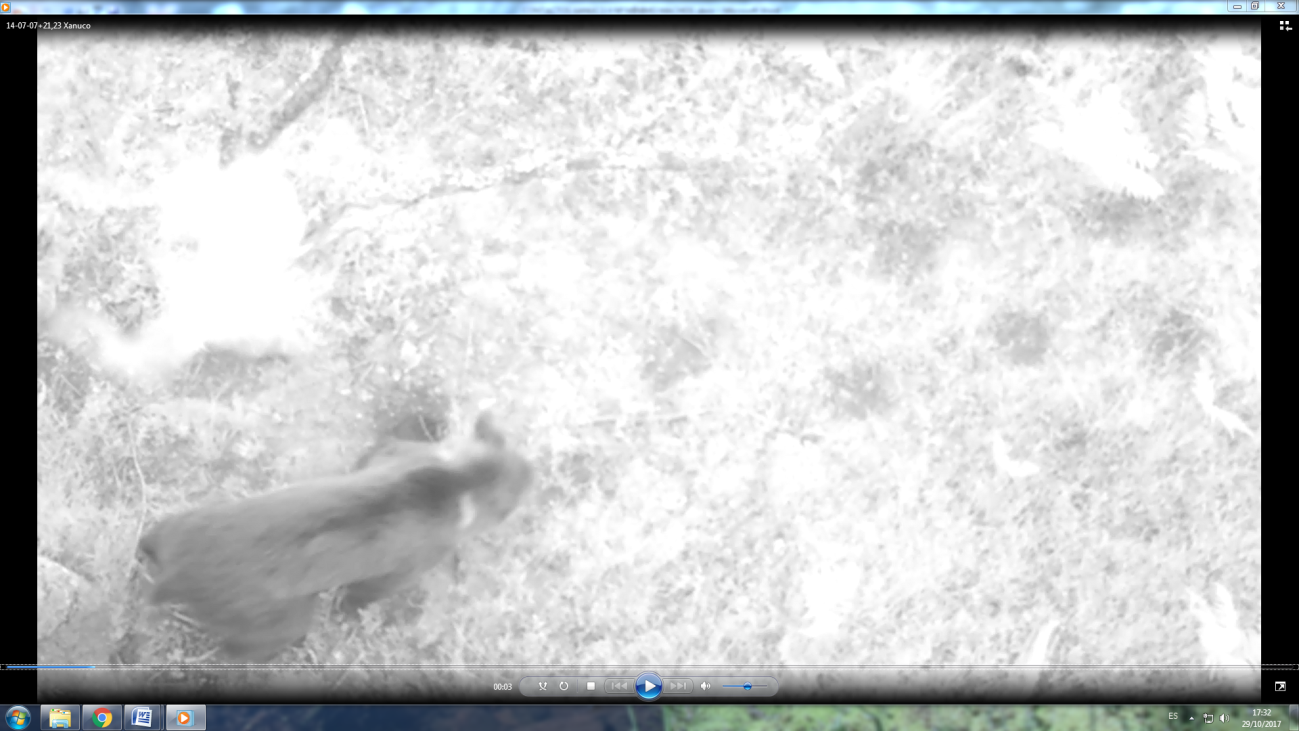

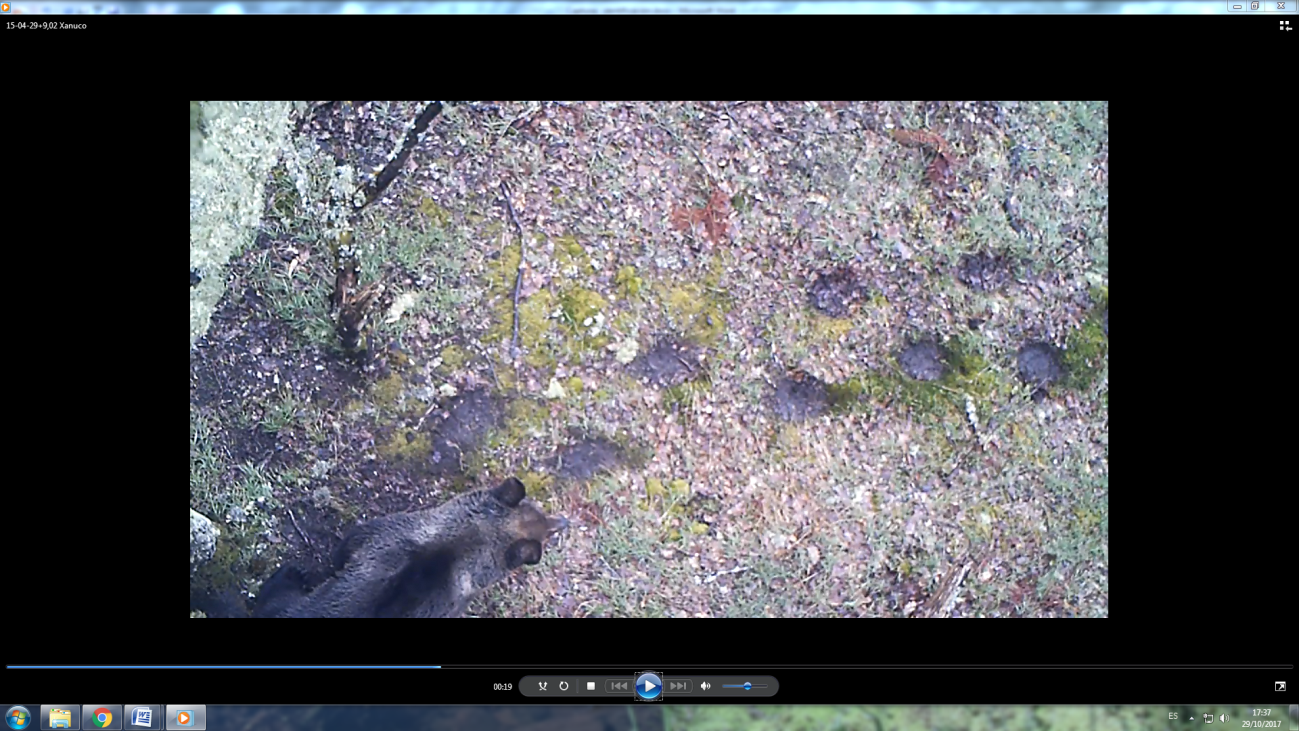


*F1 (Güesi)*

An adult female at the end of the study period, probably born in 2008 in the surroundings of the study area. We have never seen her with cubs until present, although she has participated in several mating events in the last years. Body color is light brown with extensive grizzled shadings, and wears very conspicuous golden neck markings. The marks of the right side are quite big and rhomboidal with a dark small spot at the center, and extended through the nape. The mark of left side is unconnected with the nape, with a kind of long “bone” shape.

**
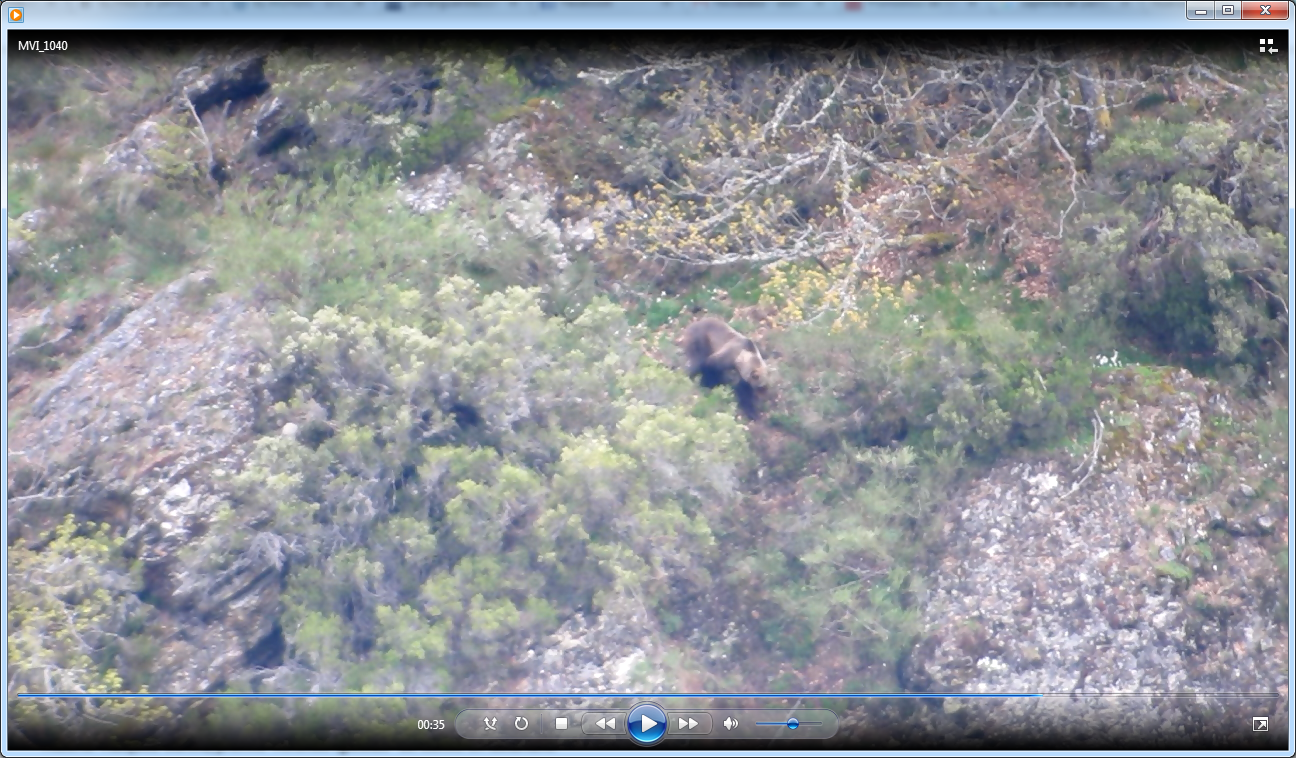
**
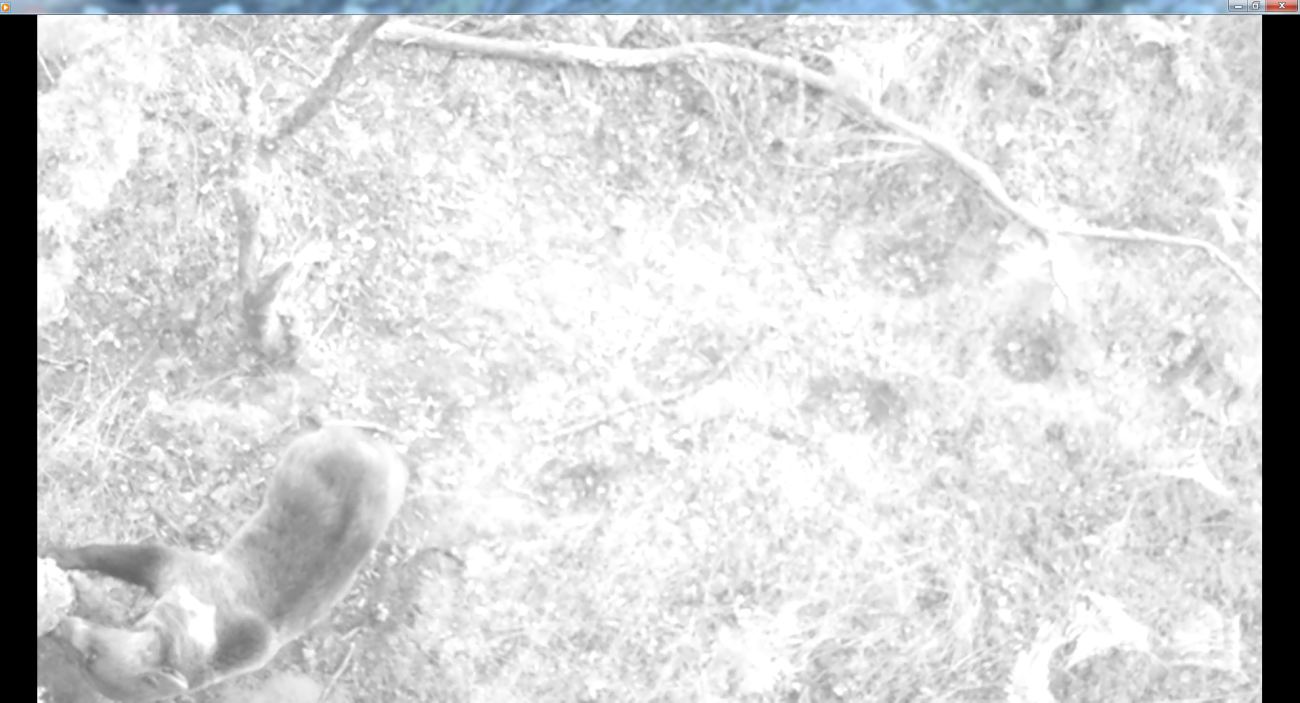

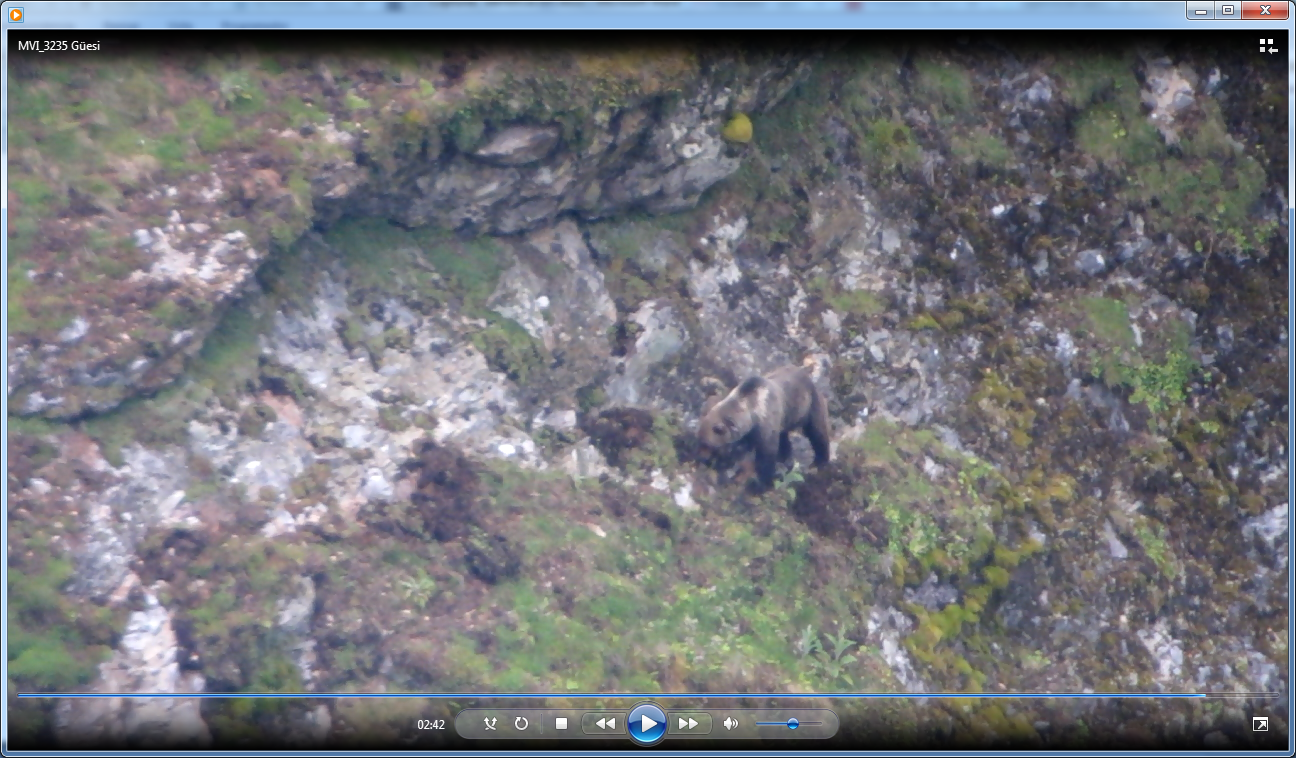

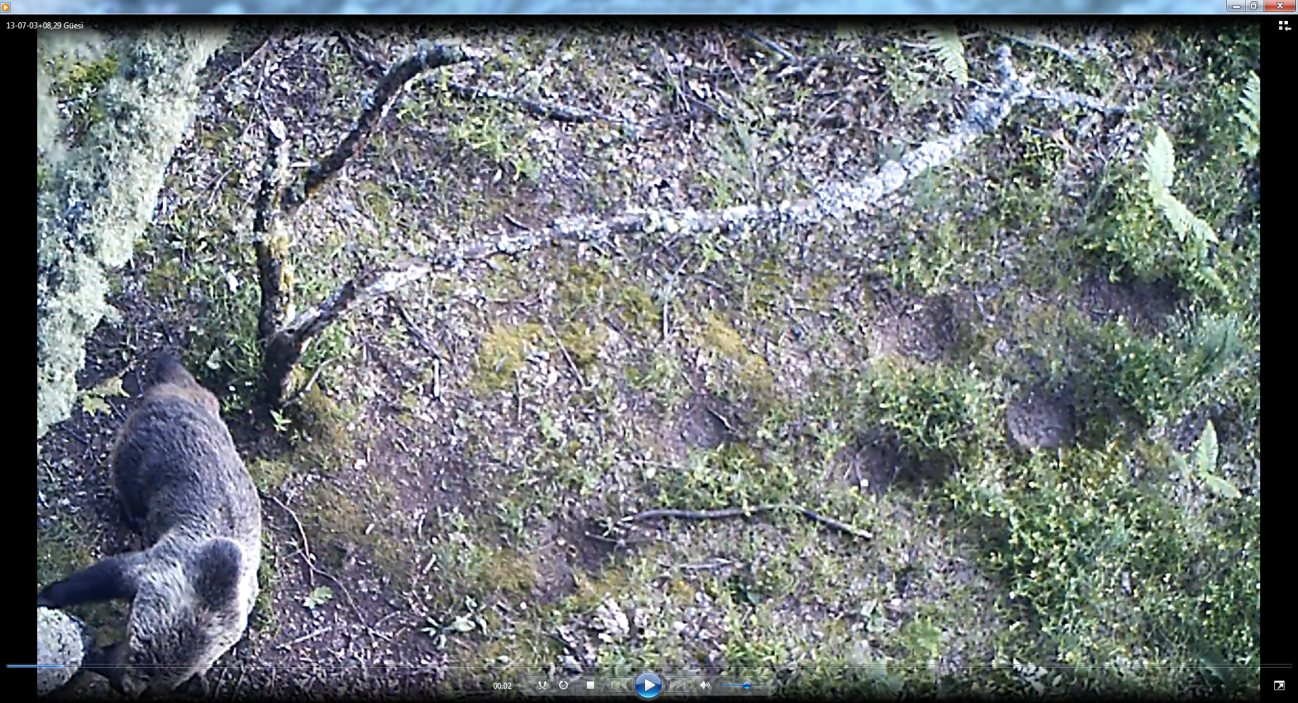

Supplement: Supplemental Information 14 [file peerj-09-10447-s014.docx]
